# Experimental characterization of the human non-sequence-specific nucleic acid interactome

**DOI:** 10.1186/gb-2013-14-7-r81

**Published:** 2013-07-31

**Authors:** Gerhard Dürnberger, Tilmann Bürckstümmer, Kilian Huber, Roberto Giambruno, Tobias Doerks, Evren Karayel, Thomas R Burkard, Ines Kaupe, André C Müller, Andreas Schönegger, Gerhard F Ecker, Hans Lohninger, Peer Bork, Keiryn L Bennett, Giulio Superti-Furga, Jacques Colinge

**Affiliations:** 1 CeMM Research Center for Molecular Medicine of the Austrian Academy of Sciences, Lazarettgasse 14, AKH-BT 25.3, 1090 Vienna, Austria; 2 Current address: IMP - Research Institute of Molecular Pathology, Dr. Bohr-Gasse 7, 1030 Vienna, Austria; 3 Current address: Haplogen GmbH, Campus Vienna Biocenter 5, Dr. Bohr-Gasse 7, 1030 Vienna, Austria; 4 Structural and Computational Biology Unit, EMBL - European Molecular Biology Laboratory, Meyerhofstraße 1, 69117 Heidelberg, Germany; 5 Current address: IMP/IMBA Bioinformatics Department, Dr. Bohr-Gasse 7, 1030 Vienna, Austria; 6 Current address: Boehringer Ingelheim RCV GmbH & Co KG, Doktor-Böhringer-Gasse 5-11, 1121 Vienna, Austria; 7 Department of Medicinal Chemistry, University of Vienna, Althanstraße 14, 1090 Vienna, Austria; 8 Institute of Chemical Technologies and Analytics, Vienna University of Technology, Getreidemarkt 9/164, 1060 Vienna, Austria

## Abstract

**Background:**

The interactions between proteins and nucleic acids have a fundamental function in many biological processes, including gene transcription, RNA homeostasis, protein translation and pathogen sensing for innate immunity. While our knowledge of the ensemble of proteins that bind individual mRNAs in mammalian cells has been greatly augmented by recent surveys, no systematic study on the non-sequence-specific engagement of native human proteins with various types of nucleic acids has been reported.

**Results:**

We designed an experimental approach to achieve broad coverage of the non-sequence-specific RNA and DNA binding space, including methylated cytosine, and tested for interaction potential with the human proteome. We used 25 rationally designed nucleic acid probes in an affinity purification mass spectrometry and bioinformatics workflow to identify proteins from whole cell extracts of three different human cell lines. The proteins were profiled for their binding preferences to the different general types of nucleic acids. The study identified 746 high-confidence direct binders, 139 of which were novel and 237 devoid of previous experimental evidence. We could assign specific affinities for sub-types of nucleic acid probes to 219 distinct proteins and individual domains. The evolutionarily conserved protein YB-1, previously associated with cancer and drug resistance, was shown to bind methylated cytosine preferentially, potentially conferring upon YB-1 an epigenetics-related function.

**Conclusions:**

The dataset described here represents a rich resource of experimentally determined nucleic acid-binding proteins, and our methodology has great potential for further exploration of the interface between the protein and nucleic acid realms.

## Background

Interactions between proteins and nucleic acids play a pivotal role in a wide variety of essential biological processes, such as transcription, translation, splicing, or chromatin remodeling, defects in which can cause multiple diseases [1]. Transcription factors that recognize specific DNA motifs constitute only part of the nucleic acid-binding proteins (NABPs), which also include less sequence-specific interactors.

The global identification of sequence-specific NABPs has so far been achieved through various approaches, such as chromatin immunoprecipitation (ChIP) in combination with either microarrays (ChIP-chip) [2-5] or sequencing technology (ChIP-seq) [6-8] as well as protein-binding microarrays [9] and protein arrays [10]. The rapid development of current proteomic technologies has opened new avenues for performing unbiased proteome-wide investigations of NABPs by affinity purification. An in-depth screen of the yeast chromatin interactome [11] was performed by applying the modified chromatin immunopurification (mChIP) approach [12], revealing several multi-protein chromatin complexes. Other researchers have employed mass spectrometry (MS) approaches to study specific aspects of protein-nucleic acid interactions. For instance, Mann and colleagues [13] demonstrated the power of such techniques by identifying interactors of functional DNA elements. Using synthetic DNA oligonucleotides, DNA sequence-specific-binding proteins and proteins that preferably interact with CpG islands were found. The same group subsequently adapted this method to RNA elements [14]. Recently, mRNA-binding proteins were surveyed by covalent UV crosslinking and affinity purification followed by MS analysis in HeLa cells [15]. This work identified 860 high confidence mRNA-protein interactions including 315 proteins not known before to bind mRNA, thereby illustrating the power of such approaches. The dataset provided new insight into the structural properties of mRNA-binding proteins, such as being enriched for short repetitive amino acid motifs and highly intrinsically disordered.

In this study, we present the first large-scale effort to map human NABPs with generic classes of nucleic acids. Using synthetic DNA and RNA oligonucleotides as baits and affinity purification (AP)-MS methods we previously applied to unravel new immune sensors of pathogen-derived nucleic acids [16,17], we performed pulldown experiments in three cell lines that yielded greater than 10,000 protein-nucleic acid interactions involving more than 900 proteins. Analysis of this rich dataset allowed us to identify 139 new high confidence NABPs, to provide experimental evidence for another 98 proteins whose NABP status had only been inferred computationally, and to determine the significant preferential affinity of 219 NABPs for different subtypes of nucleic acids, thereby complementing existing knowledge greatly. The dataset we obtained provides many entry points for further investigations, which we illustrate by proposing new functions for already characterized as well as uncharacterized proteins and domains. All the interaction data are available to the research community.

## Results and discussion

### Bait design

**Figure 1:**
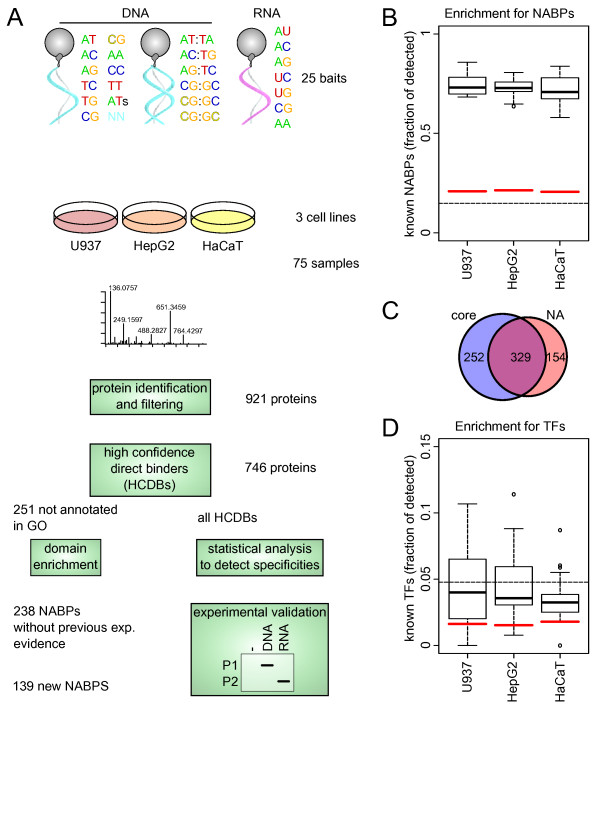
**Identification of nucleic acid binding proteins (NABPs) in human cell lines**. **(a) **Outline of the experiments and analyses performed. GO, Gene Ontology. **(b) **The affinity purification strongly enriched the identified proteins from the known NABPs (individual pulldowns summarized in the boxes) compared to human Swiss-Prot content (dashed line) and the three cell line core proteomes (red lines). **(c) **Comparison of the known NABPs in the union of the three core proteomes and all the affinity purification experiments (isoforms were collapsed for better comparability). **(d) **Proportion of annotated transcription factors (TFs) present in the core proteomes (red lines), the purifications (boxes), and Swiss-Prot (dashed lines) compared.

The diversity of all possible nucleic acid sequences that can be present in a human cell is virtually infinite and, to reduce the complexity for a general mapping of protein-nucleic acid interactions, we decided to design generic nucleic acids as baits that would capture essential differences between nucleotides. We opted for the synthesis of baits containing all possible dinucleotide combinations comprising single-stranded RNA (ssRNA), single-stranded DNA (ssDNA) and double-stranded DNA (dsDNA) (Figure 1a). The use of synthetic oligonucleotides allowed us to control bait sequences and concentrations. All the baits were 30 nucleotides in length and contained two nucleotides only in a one-to-one ratio. The choice of the actual dinucleotide pattern resulted from a maximization of the minimum free energy across all possible dinucleotide patterns using the ViennaRNA package [18] to minimize secondary structure formation. This approach was chosen to circumvent an additional layer of complexity introduced by possible secondary structures, which would have otherwise caused an explosion in the number of nucleotides to consider. To identify proteins binding to epigenetic modifications, we synthesized additional cytosine-methylated analogues of the CG-DNA oligonucleotides. Furthermore, we included several mononucleotide oligos and an ssDNA oligo with random nucleotide composition. The final set of baits comprised 25 oligonucleotides (Supplementary Table S1 in Additional file 1) and the symmetric experimental design (Figure 1a) guaranteed that differential binding of the interacting proteins would be solely due to differences in nucleotide composition. To increase the coverage of the human proteome, we performed the AP-MS experiments with whole cell lysates from cell lines derived from the three germ layers: U937 (lymphoma, mesoderm), HepG2 (liver carcinoma, endoderm), and HaCat (keratinocyte, ectoderm). To identify proteins that would bind to the streptavidin matrix - but not to the baits - we performed affinity purifications using the uncoupled matrix with each cell lysate. In total, we analyzed 78 biological samples. The synthetic oligonucleotides were coupled to a matrix by a 5' biotin moiety and used to purify NABPs from the biological samples and the enriched proteins were subsequently identified by MS (Figure 1a).

### Protein identification and filtering

Altogether, the analysis of the 78 pulldown samples yielded 10,810 protein identifications; that is, on average, 140 proteins per bait, involving 952 distinct proteins. These results were obtained by imposing a stringent protein group false discovery rate of 1% (Materials and methods). To measure the achieved enrichment for NABPs, we compared whole cell lysate proteomes acquired with the same MS technology, which we named core proteomes and published previously [19], with the enriched samples. We found that an average of 21% of proteins in the core proteomes were annotated as NABPs in Gene Ontology (GO) [20], and in the enriched samples this proportion increased to more than 70% (Figure 1b). Among the known NABPs identified in the affinity purifications, 154 were not identified in the core proteomes, indicating that our experimental approach is not limited to rather abundant proteins. Conversely, 252 out of 581 known NABPs observed in the core proteomes were not identified in the pulldowns, thereby suggesting that these NABPs recognize sequence-specific nucleic acids or patterns not present among the baits (Figure 1c). With respect to transcription factors, the purification protocol provided a modest enrichment over the core proteomes only (Figure 1d). This was not surprising since transcription factors are usually lowly abundant [21] and bind to specific sequence elements.

The physical detection of interacting proteins by AP-MS can also result in the identification of abundant non-interacting entities. To circumvent this problem, we exploited negative control pulldowns where we identified 72 proteins, 41 of which were well-known abundant NABPs that should be retained in the final dataset - for example, histones and ribosomal proteins. Therefore, we did not subtract the negative controls directly but required that identified proteins were either absent from the negative controls or were detected with at least five times more spectra in the real samples (Supplementary Table S2 in Additional file 1). This filter reduced the number of distinct proteins to 921 entities, which included 25 out of the 41 abundant NABPs mentioned above.

**Figure 2:**
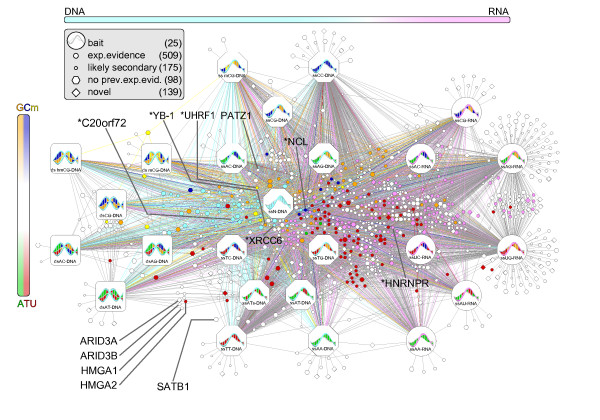
**Overview of the nucleic acid interactome**. Baits are indicated by large nodes. Nucleotide composition of the baits and preferential affinity of proteins are color coded according to Figure 1a. In case of multiple substrate preferences for a single protein, only the most significant one is reported. Interacting proteins are split into four groups (known with experimental evidence, likely secondary, no previous experimental evidence, and novel) based on public annotations and interaction databases. Selected proteins that have been experimentally validated (preceded by an asterisk) or are well known are indicated with a color code indicating their inferred or known preferential affinity (dual affinities were arbitrarily assigned one color).

Another important feature of purification-based protocols is that partial or entire protein complexes are retrieved - that is, a NABP that interacts directly with the bait may lead to the co-purification of its own protein partners that are not necessarily NAPBs. To limit this phenomenon, we used appropriate washing steps (Materials and methods) and exploited known physical protein-protein interactions collected from public repositories [22-27]. All the pulled down proteins known to physically interact with another protein annotated as a NABP in GO were considered as likely secondary binders, leaving 746 high confidence direct binders (HCDBs), which are the basis of most of our subsequent analyses. These include 139 proteins not annotated as NABP in GO and not found in data from [15] (we had a stringent requirement that data from [15] be novel rather than what was selected above a 1% q-value in the study, thus constituting novel NABPs (Supplementary Table S3 in Additional file 1). An additional 98 proteins had no previous experimental evidence indicating they are NABPs (not in [15], GO evidence code 'IEA' for electronic annotation); thus, we provide the first experimental evidence for 237 NABPs. An overview of the nucleic acid interactome is presented in Figure 2.

### A high quality dataset

**Figure 3:**
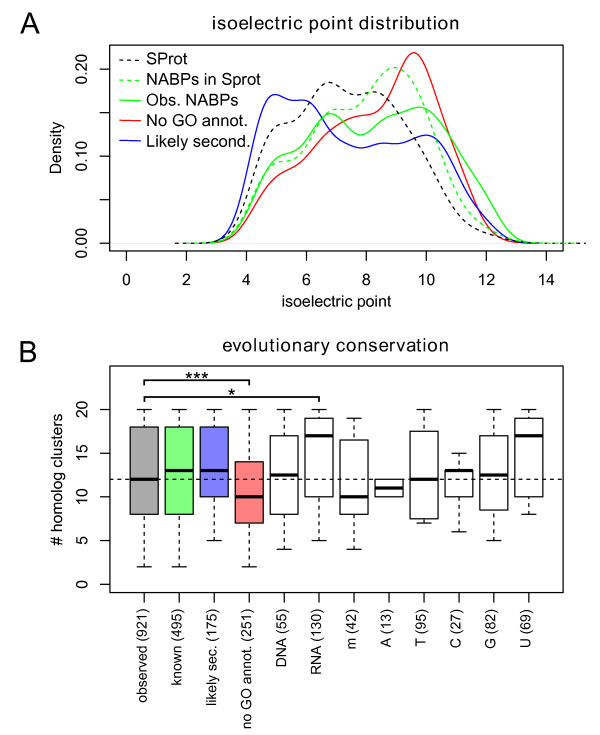
**Characteristics of the identified proteins**. **(a) **Comparison of pI distributions for the already known, and enriched NABPs in our data (solid green line) versus all Swiss-Prot human proteins (dashed black line) and known human NABPs in Swiss-Prot (dashed green line). NABPs without nucleic acid binding GO annotation (solid red line) had a more pronounced shift towards higher values, whereas the likely secondary binders had the opposite trend (solid blue line). **(b) **Sequence conservation as determined by the number of orthologs reported in Ensembl. Compared to all the enriched proteins (left gray box), the newly identified NABPs were significantly less evolutionarily conserved (*** *P *< 0.005, KS test) and the RNA-specific NABPs more conserved (* *P *< 0.05, KS test). The other groups showed no significant difference. RNA-specific NABPs contain many ribosomal proteins, which explain the average higher conservation.

We performed several analyses to assess the quality of the data obtained. NABPs are known to be enriched for positively charged proteins and we therefore compared the distribution of the isoelectric points (pI) of several reference protein sets with our experimental results. Compared to all the human proteins described in Swiss-Prot, Swiss-Prot human NABPs were indeed shifted towards higher pI values (*P *< 6.5E-81, Kolmogorov-Smirnov test; Figure 3a). The same trend was more pronounced for the proteins we identified that were already annotated as NABPs (*P *< 4.7E-17, KS test). The 251 identified proteins that were not annotated as NABPs in GO featured an even stronger shift and were nicely contrasted by the likely secondary binders.

The number of known NABPs found in each cell line (Supplementary Figure S1 in Additional file 1) varied modestly, thus showing experimental reproducibility, and the GO analysis of the molecular functions of HCDBs identified RNA- and DNA-related terms almost exclusively (Supplementary Table S4 in Additional file 1).

We also found that the 251 NABPs not annotated by GO evolved more recently, indicated by a smaller number of orthologs found in Ensembl [28] (*P *< 2.6E-4, KS test; Figure 3b). This observation is compatible with classical genome annotation methods that transfer protein functional annotations by homology and are thus more likely to fail on less similar protein sequences.

### Nucleotide specificity

The synthetic bait design allowed us to correlate differential protein abundances across the samples against the composition of the bait, thereby inferring prey protein binding specificities, that is, strong preferences for certain subtypes of nucleic acid. To systematically determine these affinity preferences required a tailored statistical test that relied on relative protein abundance reflected by the number of spectra that supported the protein identification (spectral count; Materials and methods). Application of the statistical test to proteins in the HCDB group to query for preferential affinity for DNA, RNA, adenine (A), thymine (T), cytosine (C), guanine (G), uracil (U), and methylated cytosine (mCG) resulted in 513 significant preferential affinities by 219 distinct proteins (*P *< 0.05; Figure 2; Supplementary Table S5 in Additional file 2); that is, some NABPs had multiple preferences.

**Figure 4:**
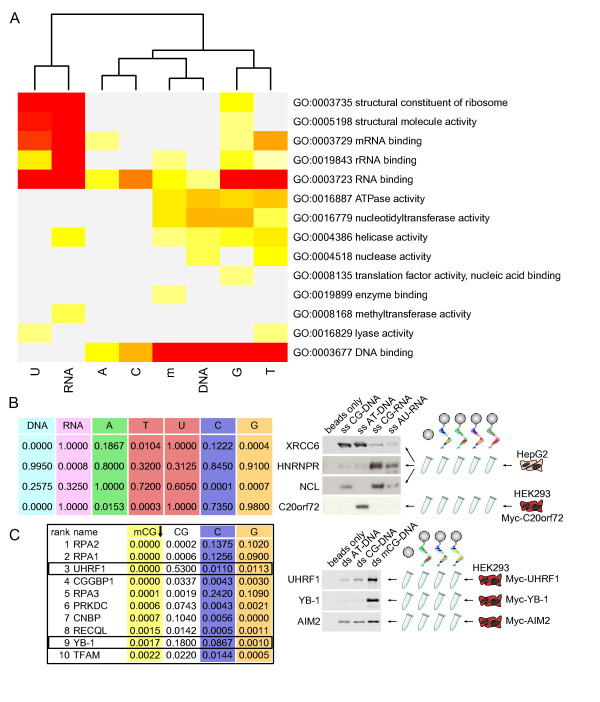
**Validation of preferential affinity**. **(a) **GO molecular function term significance in the various sets of proteins inferred to bind preferentially one or several subtypes of nucleic acids. We observe the clear separation between molecular functions enriched in inferred DNA- and RNA-binding proteins. Color log-scale: red = *P *< 1E-15, light yellow = *P *< 0.01, gray = *P *≥ 0.01. **(b) **Examples of affinity preferences of selected NABPs represented by *P*-values in the statistical analysis (table on left) and western blots in the experimental validation (right). We note the strong agreement between preferred versus non-preferred affinities in the statistics and the blots. (C20orf72 was purified with a Myc tag in HEK293 cells instead of a specific antibody in HepG2 cells.) **(c) **Methylation specificity usually correlates with CG specificity, but UHRF1 and YB-1 were specific to mCG only in the statistical analysis (see reported *P*-values in the table on the left). Experimental validation confirmed their specificity (right); AIM2 was used as a DNA-binding non-specific control.

To determine the success rate of the test statistics, we estimated true and false positive rates (TPR and FPR) on the basis of known DNA- and RNA-binding proteins (GO annotations and data from [15] additionally for RNA). We found that the inferred DNA preferential affinities had a TPR of 23.0% and a FPR of 2.8%, whereas inferred RNA preferential affinities had a TPR of 18.7% and a FPR of 1.6%. This validated the reliability of our predictions as well as the accuracy of the estimated *P*-values from our tailored statistical test. It further indicated medium sensitivity and closer inspection showed that missed specificities suffered from limited spectral counts, that is, experimental sensitivity (Supplementary Figure S2 in Additional file 1). In total, we inferred 130 RNA, 55 DNA, 13 adenine, 95 thymine, 27 cytosine, 82 guanine, 69 uracil, and 42 methylated cytosine significant preferential affinities. GO enrichment analyses further confirmed the accuracy of this procedure by associating inferred DNA-specific proteins and inferred RNA-specific proteins with DNA- and RNA-related GO terms, respectively (Figure 4a; Supplementary Figures S3 and S4 in Additional file 1). This can also be observed at an individual protein level in Supplementary Table S5 in Additional file 2 where DNA-specific proteins are dominated by well known DNA-associated proteins such as DNA repair enzymes, histones, and so on. The same is true for RNA-specific proteins (ribosomal proteins, translation initiation factors, and so on).

In the case of specificities for CpG methylated cytosines (mCG), the most abundant form of methylation in nature, the methylated oligos formed a subset of the C- and G-containing oligos. CG-specific proteins were thus frequently detected as methylation-specific in the pulldowns. To dissect this correlation, we computed an additional specificity for unmethylated CG oligos, which could be used to distinguish methyl-specific proteins from proteins with general CG specificity (Figure 4c, column CG). Comparing these specificities, we identified UHRF1 (ICBP90) as a methylation-specific protein, which was previously shown to recognize methylated cytosines [29] and hence served as validation. A new protein with high specificity for methylated CG baits was YB-1 (see below).

**Figure 5:**
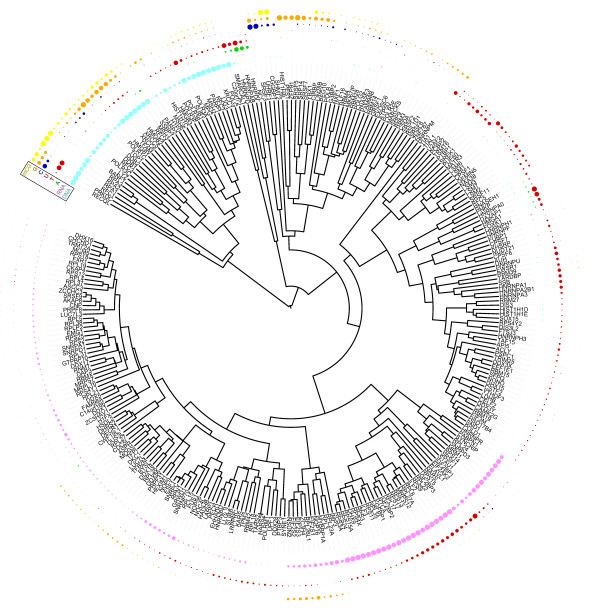
**The 219 proteins that were assigned a binding preference for at least one nucleic acid subtype have been clustered to reflect similarities in preferences (affinity fingerprint)**. Most protein families show similar preferences. In contrast, H1FX was found to be RNA-specific as opposed to the family members H1F0 and HIST1H1C, which were DNA-specific.

A global tree representation of the inferred preferential affinities was created on the basis of the *P*-values for each type of nucleic acid probe (Figure 5). In general, we observed that protein families tended to form clusters in the tree but substrate specificity transfer to paralogs was not always valid, which is another illustration of the difficulty of assigning protein functions solely by sequence homology.

### Validation

To verify our predictions, we decided to perform experimental validations. The number of preferential affinities tested was maximized by selecting four proteins for which several nucleic acid subtypes were enriched with strong *P*-values in each case. We chose X-ray repair cross-complementing protein 6 (XRCC6, also known as Ku70), predicted as DNA-, thymine- and guanine-specific; heterogeneous nuclear ribonucleoprotein R (hnRNPR), predicted as RNA-specific; nucleolin (NCL), predicted as cytosine- and guanine-specific; and the uncharacterized protein C20orf72, predicted as DNA-, adenine-, and thymidine-specific. We repeated affinity purifications using cell lysates from HepG2 cells with a limited number of nucleic acid baits and assessed binding by immunoblotting for these candidate interactors. Since no antibody was available for C20orf72, we cloned a tagged form and expressed it in HEK293 cells, a widely used human embryonic kidney cell line, as these are more amenable to transfection. Using western blotting (Figure 4b), we observed that XRCC6 clearly preferred DNA with no difference between AT- and CG-rich substrates, which is compatible with T and C affinity as predicted. HNRNPR showed a clear affinity for RNA according to the prediction. NCL bound to CG-rich substrates, both DNA and RNA, which is in agreement with the computational analysis. Finally, C20orf72 had an exclusive affinity for AT-rich DNA as inferred. We hence obtained results matching the computations in terms of both inferred preferential affinities and absence of preferences accurately.

Additional evidence of correct statistical analysis was provided by proteins whose selectivity towards nucleotide composition is well documented. The CGG triplet repeat-binding protein 1 (CGGBP1, UniProt Q9UFW8) was found to have strong DNA and C- and G-rich nucleotide preference (Supplementary Table S5 in Additional file 2), which recapitulates what is known about its substrate preferences [30]. The same is true for the high mobility group protein HMG-I/HMG-Y (HMGA1, P17096), found to prefer A- and T-rich nucleotides [31].

HMGA1 contains an AT hook domain that is also present in two additional NABPs we identified but not predicted to have a significant preference for A- and T-rich oligos. These proteins are the POZ-, AT hook-, and zinc finger-containing protein 1 (PATZ1, Q9HBE1) and the high mobility group protein HMGI-C (HMGA2, P52926). Checking their full spectral count data, we observed that they were only expressed in HepG2 cells (Table 1). HMGA2 was clearly detected as preferentially binding only dsDNA and ssDNA AT-rich nucleotides, whereas PATZ1 was found to preferentially bind only generic ssDNA with low spectral count. These two examples illustrate the impact of limited MS sensitivity on probably lowly expressed proteins and its consequence on the data analysis (discussed in the 'Nucleotide specificity' section above). To have a stringent test for preferential affinity, we imposed detection in several cell lines but - with higher risk - compositional preference could be mined more broadly. Following this route, we queried our data for proteins detected in at least one cell line and with more than eight spectra with an AT-rich bait and zero spectra with CG-rich baits. We found another three AT-rich nucleotide-specific proteins (Table 1): the AT-rich interactive domain-containing proteins 3A and 3B (ARID3A, Q99856; ARID3B, Q8IVW6) and the DNA-binding Special AT-rich sequence-binding protein 1 (SATB1, Q01826).

**Table 1:** Spectral counts of substrate composition-specific nucleic acid-binding proteins

		U937							HepG2							HaCat						
				
		ssDNA			dsDNA				ssDNA			dsDNA				ssDNA			dsDNA			
							
AC	Name	*dN*	dCdGm	dAdTs	dCmdG:dCmdG	dCdG:dCmdG	dCdG:dCdG	dAdT:dAdT	*dN*	dCdGm	dAdTs	dCmdG:dCmdG	dCdG:dCmdG	dCdG:dCdG	dAdT:dAdT	*dN*	dCdGm	dAdTs	dCmdG:dCmdG	dCdG:dCmdG	dCdG:dCdG	dAdT:dAdT
Q9UFW8	CGGBP1	***0***	23	0	6	5	7	0	***0***	0	0	4	10	13	0	***0***	0	0	0	0	1	0
P17096	HMGA1	***0***	0	12	0	0	0	4	***0***	0	10	0	0	0	14	***0***	0	2	0	0	0	0
P52926	HMGA2	***0***	0	0	0	0	0	0	***0***	0	4	0	0	0	10	***0***	0	0	0	0	0	0
Q9HBE1	PATZ1	***0***	0	0	0	0	0	0	***2***	0	0	0	0	0	0	***0***	0	0	0	0	0	0
Q8IVW6	ARID3B	***0***	0	0	0	0	0	0	***0***	0	2	0	0	0	9	***0***	0	0	0	0	0	0
Q99856	ARID3A	***0***	0	0	0	0	0	0	***0***	0	10	0	0	0	59	***0***	0	0	0	0	0	0
Q01826	SATB1	***0***	0	0	0	0	0	9	***0***	0	0	0	0	0	0	***0***	0	0	0	0	0	0

Nucleotide subtypes not displayed had zero spectral count (dN indicates ssDNA random sequences). AC=UniProt accession code.

To experimentally evaluate YB-1 cytosine methylation specificity, we expressed UHRF1 and YB-1 as tagged forms in HEK293 cells and assessed methylation-specific nucleic acid binding comparing CG ds DNA with mCG dsDNA bearing abundant cytosine methylation. We also included AT dsDNA to exclude the potential CG bias mentioned above. AIM2, an immune sensor for foreign DNA with no known nucleic acid-binding specificity [16], was included as additional control. While AIM2 was found to bind to all DNA baits alike, UHRF1 showed a strong preference for methylated DNA (Figure 4b). YB-1 was highly specific for methylated DNA as well and was not detectable in the non-methylated DNA samples (Figure 4c). On a genome-scale, we obtained supplementary evidence of YB-1 affinity by performing a ChIP-seq experiment in HEK293 cells (Materials and methods). Intersection of YB-1 interaction sites (ChIP-seq peaks) with four HEK293 reduced representation bisulfite sequencing datasets [32] from ENCODE showed significant enrichment for methylated CGs (*P *< 0.05, KS test) in three out of the four samples (Supplementary Figures S5 and S6 in Additional file 1).

Uracil bases present in RNA but not in DNA and thymine bases present in DNA but not in RNA provide another means of global validation. Most NABPs preferring uracil should not have any affinity for T-rich oligos and vice versa and, indeed, in our calculations (Supplementary Table S5 in Additional file 2) we observe very little overlap (5 proteins) among the T-specific proteins (35) and the U-specific proteins (86) (*P *< 6.1E-23, hypergeometric test).

### Limitations of the dataset

The necessary selection of oligonucleotides of low sequence complexity and devoid of secondary structure to maintain the number of baits within a reasonable range certainly had an impact on the NABPs that we could actually identify.

Low sequence complexity has the potential to induce the identification of numerous abundant proteins that could have low affinity for nucleic acids - for example, sequence-specific NABPs that would retain low nucleic acid affinity for some of the baits we used. Although this phenomenon certainly exists, convergent and independent observations show that it does not contribute to an important level. In the 'Protein identification and filtering' section we noted that, while the proportion of known NABPs rose from 21% in the core proteomes to 70% in the pulldowns, 252 NABPs of the core proteomes - hence abundant - were not identified in the affinity-purified samples, thus indicating affinity purification specificity. Extending this analysis to transcription factors, which are sequence-specific predominantly, we observed that general NABPs were much more enriched in pulldowns compared to transcription factors (Figures 1b,d), further showing the absence of a strong nucleic acid low affinity-driven bias on this class of proteins. Moreover, carefully realized pulldown experiments with non-specific interactions removed (for example, comparing with proper negative controls as was done in this study) have a long history of revealing relevant protein interactions - for example, with oligonucleotide baits [16,17]. In line with this, inspection of Supplementary Table S5 in Additional file 2 for DNA- or RNA-specific NABPs reveals numbers of well known DNA- and RNA-associated proteins with a functional role.

The lack of secondary structures that might be required for binding certain proteins is likely to have limited our sensitivity. It is difficult to evaluate the extent of this phenomenon precisely but the recently published mRNA interactome [15] provided us with the opportunity to compare large and unbiased datasets, with and without secondary structures, obtained via roughly comparable technology platforms. We assumed that the mRNA interactome captured the majority of secondary structure-dependent interactions since highly specific covalent UV crosslinking was applied. It unraveled 315 novel mRNA binding proteins whereas we found 247 novel NABPs considering all the baits (the 139 novel proteins we claim plus overlap with the mRNA interactome otherwise removed). Considering just RNA baits, we identified 177 novel interactions. In terms of totals reported, the mRNA interactome was composed of 860 mRNA binders and we obtained 746 NABPs for all the baits; 557 for RNA baits only. One could thus estimate a roughly two-fold reduction in sensitivity, showing clearly that a large reduction in sensitivity (for example, ten-fold) is not supported by the comparison of these two datasets. Indeed, the large overlap between the 860 proteins of the mRNA interactome and the 557 we identified via RNA baits is very significant (301 proteins; *P *< 3.3E-91, hypergeometric test).

### Domain analysis

**Figure 6:**
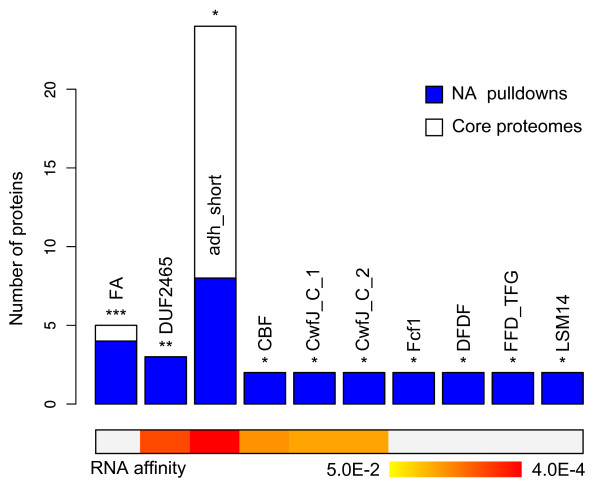
Domains enriched among the nucleic acid high confidence direct binders (HCDBs) lacking known nucleic acid domains (***P < 0.005, **P < 0.01, *P < 0.05; binomial test with Benjamini-Hochberg correction). These domains are likely to confer nucleic acid affinity. Remarkably, we identified in our pulldowns all the human proteins harboring the DUF2465, CwfJ_C_1 and 2, and Fcf1 domains with specific peptides (two out of three for CBF/Mak21). By combining individual protein preferential affinities for subtypes of nucleic acids (Supplementary Table S5 in Additional file 2), we could determine domain RNA preference *P*-values (color scale at the bottom on the basis of the *P*-value logarithms; subtypes other than RNA did not yield significant results).

The identification of novel NABPs offered a unique opportunity to recognize previously unknown nucleic acid binding by certain domains. We used Pfam [33] as a domain database and considered the proteins in the HCDB group devoid of a domain known to bind nucleic acids, which left us with 236 proteins. Using the U937, HepG2, and HaCat core proteomes and all the proteins found in the pulldowns as background, we found ten domains to be significantly enriched (*P *< 0.05, binomial, Benjamini-Hochberg (BH) corrected) and could infer RNA preferences for five of them (Figure 6; Supplementary Table S6 in Additional file 1). Among the ten enriched domains we found the well conserved domain of unknown function DUF2465. All three human proteins harboring this domain (FAM98A, B, C) were identified in our pulldowns and DUF2465 was assigned a preference for RNA, which is well supported by previous identifications of FAM98A as a mRNA binder [15] and FAM98B as a component of the tRNA-splicing ligase complex [34].

Four proteins whose functions are poorly understood harbored both the FERM and FERM adjacent (FA) domains: the Band 4.1-like proteins 1, 2, and 5; and the FERM, RhoGEF and pleckstrin domain-containing protein 1. The FERM domain is known to bind membrane proteins and lipids. It is found in proteins at the interface of the cytoskeleton and the plasma membrane that reorganize the membrane microstructure and coordinate the disposition of signaling and cell adhesion complexes. The FA domain is present in a subset of FERM-containing proteins and is believed to regulate the FERM domain activity [35]. Our data thus suggest a possible FERM modulation influenced by nucleic acid binding.

**Figure 7:**
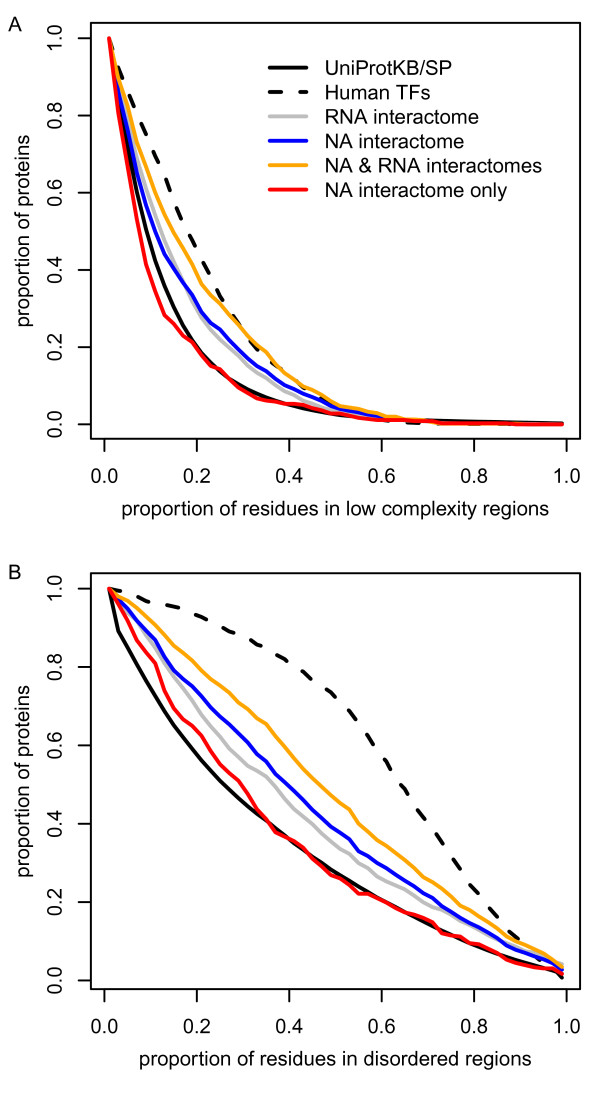
**Low complexity and disordered regions**. **(a) **Compared to an average human protein, the proteins found in the nucleic acid (NA) interactome contained more residues in low complexity regions (*P *< 1.7E-11, KS test), a bias similar to what was observed for the mRNA interactome. This bias is augmented for proteins in both interactomes as opposed to proteins in the nucleic acid interactome only (mRNA interactome subtracted), which are mostly non-sequence-specific DNA binders. **(b) **The same biases are observed for disordered regions. TF, transcription factor.

Protein sequence analysis of the mRNA interactome [15] revealed an overrepresentation of unstructured and low complexity segments among the identified mRNA binding proteins. We performed the same analysis to compare with NABPs found in this study. We found a very similar bias towards the presence of low complexity and disordered regions (Figure 7), which we decomposed into proteins found in both studies and proteins found in ours only. The shared proteins further increased this bias, which is coherent with the design of our baits aimed at being non sequence-specific. On the contrary, the proteins unique to our data followed the average human protein trend. These proteins are likely to bind DNA and we thus wanted to assess whether transcription factors might be the cause of this inversed result, but it was not the case since human transcription factors are actually very rich in low complexity and disordered regions (Figure 7). Moreover, their contribution to the datasets is modest: 3.2% of the mRNA interactome (dual DNA/mRNA binding or false positives in one of the two studies), 4.9% of our data, 2.9% in both, and 7.9% in our data only. Therefore, we conclude that non-sequence-specific proteins binding DNA, which are not transcription factors, do interact with nucleic acid chains through an interface that is more constrained in its geometry than proteins binding mRNA.

### Intersecting NABPs with human diseases

We searched all the novel NABPs discovered in this study against the Genetic Association Database [36] and found that 30 of them are the products of genes involved in several pathologies (Supplementary Table S7 in Additional file 1). Given the importance of DNA or RNA metabolism deregulation in many diseases, these new insights might contribute to the understanding of disease etiology or dynamics. For instance, we note that six Alzheimer's disease-related proteins can bind nucleic acids, which could provide additional links with stress granules in Alzheimer's disease and other neuropathologies [1,37,38].

YB-1 affinity for methylated cytosines was an intriguing finding that we wanted to explore in more detail. YB-1 is a multifunctional cold shock domain-containing protein known to have an affinity for both DNA and RNA and to be involved in nucleic acid stabilization, transcription, and translation [39,40]. YB-1 plays a role in environmental stress response and is over-expressed in tumors [41,42], where it is associated with multiple drug resistance and poor prognosis [41,43,44] - for example, by increasing the expression of MDR1 and P-glycoprotein [43], and upon translocation from the cytoplasm to the nucleus following S102-phosphorylation [42,43]. To understand the transcriptional impact of YB-1 caused by its binding affinity, we decided to map YB-1 ChIP-seq peaks to the nearest genes (maximum 5,000 bp distance). We found 206 genes (Supplementary Table S8 in Additional file 3) and the KEGG (Kyoto Encyclopedia of Genes and Genomes) [45] pathway analysis best hit was a weak association with cancer (*P *< 0.052, hypergeometric test, no BH correction). To test this trend, we exploited public protein interactions [22-27] to identify a subnetwork containing 73 of the targeted genes (Supplementary Figure S7 in Additional file 1) that was strongly associated with KEGG cancer pathways (*P *< 3E-4, hypergeometric test, BH correction). This suggested a potential epigenetic component to YB-1 nuclear activity providing a complementary hypothesis for the proliferative phenotype of certain tumors in relation to YB-1 nuclear translocation.

## Conclusions

We have established a first, unbiased nucleic acid-protein interaction screen aimed at identifying NABPs on the basis of systematic and comparable experimental observations not oriented towards sequence-specific nucleic acid affinity. This screen successfully provided the first experimental evidence for 237 NABPs, 139 of which were completely novel, showing that DNA and RNA biology still include large, unexplored regions to be discovered. By exploiting the particular bait design, we could further dissect the broad nucleic acid affinity of 219 proteins into 513 significant preferences for subtypes of nucleic acids (Supplementary Table S5 in Additional file 2). The high quality of the data generated in this study is supported by experimental validation and by several additional analyses, such as characteristic pI distributions for NABPs (Figure 3b) and distinct GO term enrichments for RNA- versus DNA-specific proteins (Figure 3a; Figures S2 and S3 in Additional file 1). The limitations introduced by low sequence complexity oligonucleotide baits devoid of secondary structure were analyzed and found to impact the sensitivity of the analysis but not its quality. We also demonstrated that the sensitivity achieved was comparable with native mRNA-cross-linked protein pulldowns published recently [15].

The proposed method implements a new and integrated experimental and computational procedure. The many new NABPs and nucleic subtype preferences identified show its important discovery potential. Compared to previous methods, it retains full information about the nucleic acid bound. This aspect can be fundamental to untangle direct interactions in situations such as gene transcription where DNA and RNA molecules are physically close and protein complexes might bind both types of nucleic acids. Intersecting proteins we inferred to have strong preferential affinity for DNA (*P *< 0.01), but not for RNA, with the mRNA interactome from [15] we found: PARP1, XRCC6, XRCC5, SUB1, TFAM, SSBP1, H1F0, HMGB1, HIST1H1C, and HMGB2. These proteins are well known to bind DNA, which is nicely reported in our data (Supplementary Table S5 in Additional file 2), but were confusingly found in mRNA pulldowns, which could result in wrong annotations for uncharacterized proteins.

The main contribution of this study is to provide a rich experimental resource to the community to intersect and compare with specialized fields of research. We illustrated this great potential by discussing implications of the identified YB-1 affinity for methylated cytosines (Figure 4c; Figure S4 in Additional file 1) in cancer. Access to previously unknown nucleic acid affinities also allowed us to shed light on the function of uncharacterized domains and proteins, such as the C20orf72 protein, which was confirmed to be AT-DNA-specific in the experimental validations (Figure 4b), or the DUF2465 domain proposed to bind RNA (Figure 6). Mining our data deeper, beyond the rigorous statistical procedure identifying the 513 preferential affinities mentioned above, we could demonstrate that more correct nucleotide composition-specific interaction could be found. To which extent such *in vitro*-observed nucleic acid-protein interactions remain true *in vivo *is a natural question to ask, especially since recent reports revealed confounding binding events occurring after cell lysis [46,47]. A general answer is beyond the scope of this work as it would require a gigantic effort to functionally validate all novel interactions. Nonetheless, the same technology was at the source of fundamental discoveries in innate immunity originating from *in vitro *analyses subsequently validated *in vivo*, as illustrated by the finding of AIM2 being the inflammasome DNA-binding component [16] and IFITs being 5' triphosphate RNA binders [17]. The latter was even followed by the elucidation of the three-dimensional structure of the co-complex [48]. This shows that our data provide a rich repository for experimentally derived nucleic acid-binding proteins supporting the identification of novel protein functions or new substrate affinities.

The presented approach can be readily scaled-up by introducing additional baits and/or more sensitive MS to explore deeper nucleic acid interactomes, including in projects where different samples or experimental conditions - for example, drug treatments or viral infection - would be compared. All the protein identifications are released in Supplementary Table S9 in Additional file 4 and have been submitted to IntAct [23] as well (Materials and methods).

## Materials and methods

### Nucleic acid affinity purification

Oligonucleotides were synthesized by Microsynth (Vienna, Austria).The sense strand was biotinylated at the 5' end; the antisense strand was not modified. Double-stranded baits were annealed by heating to 80°C for 10 minutes, followed by slow cooling to 25°C. For generating the affinity resin, Ultralink immobilized Streptavidin Plus Gel (Pierce, Fisher Scientific, Vienna, Austria) was washed three times with PBS. Four nmol of nucleic acid (single or double stranded) were then added to the streptavidin resin equilibrated in PBS, followed by incubation at 4°C for 1 h on a rotary wheel to allow binding of the biotinylated oligonucleotides. Next, the resin was washed twice with PBS and twice with TAP lysis buffer (50 mM Tris, pH 7.5, 100 mM NaCl, 5% (v/v) glycerol, 0.2% (v/v) Nonidet-P40, 1.5 mM MgCl_2_, 25 mM NaF, 1 mM Na_3_VO_4 _and protease inhibitor 'cocktail' (Complete; Roche, Vienna, Austria) for the removal of unbound oligos. Cells were lysed in TAP lysis buffer. For every 4 nmol immobilized nucleic acid, 6 mg cell extract was used for nucleic acid affinity purification. Additionally, 10 µg/ml poly(I:C) (for DNA baits) or 10 µg/ml calf-thymus DNA (for RNA baits) were added as soluble competitor. Cell extracts were combined with the immobilized nucleic acids, followed by incubation for 2 h at 4°C on a rotary wheel. Unbound proteins were removed by three consecutive washes in TAP lysis buffer. Bound proteins were eluted with 300 µl 1 M NaCl.

For the validation of XRCC6, HNRNPR and NCL were detected by immunoblotting using available antibodies (AB1358, 05-620, 05-565; Millipore, Vienna, Austria). Myc-tagged C20orf72, AIM2, UHRF1 and YB-1 were overexpressed in HEK293 cells and visualized by immunoblotting using anti-Myc-IRDye800 (Rockland **Gilbertsville, PA, USA**). Bound proteins were eluted in SDS sample buffer for validation experiments.

### Liquid chromatography-mass spectrometry and data analysis

Samples were analyzed on a hybrid LTQ Orbitrap XL mass spectrometer (ThermoFisher Scientific **Vienna, Austria**) coupled to a 1200 series high-performance liquid chromatography (HPLC) system (Agilent Technologies **Munich, Germany**) with an analytical column packed with C18 material. Data generated by tandem MS were searched against the UniProtKB/Swiss-Prot database version 57.12 [49] using the Mascot [50] and Phenyx [51] search algorithms. The returned protein identifications were integrated as previously described [19] with an imposed false discovery rate of 1% on the identified protein groups. Interactions were submitted to IntAct (see Supplementary Table S10 in Additional file 5 for a list of bait IntAct identifiers).

### YB-1 ChIP-seq experiment

EST for YB-1 was cloned into pFMIG STREP-3xHA plasmid using the Gateway cloning system (Invitrogen). HEK293 cells were cultivated in DMEM (PAA Laboratories **Pasching, Austria**) supplemented with 10% fetal calf serum (Invitrogen) and antibiotics (penicillin (100 U/ml) and streptomycin (100 μg/ml)). ChIP was performed according to Valouev *et al. *[52]. Briefly, Hek-Flp-In cells were transiently transfected for 24 h with polifectamine (Invitrogen). Cells (1 × 10^8^) were crosslinked with 10% formaldehyde for 10 minutes, quenched with glycine for 5 minutes and then harvested. Cells were resuspended in LB1 buffer (50 mM Hepes pH 7.6, 140 mM NaCl, 1 mM EDTA, 10% glycerol, 0.5% NP-40, 0.25% Triton X-100) to lyse the cytoplasms and the released nuclei were washed once in LB2 buffer (10 mM Tris-HCl pH 8.0, 200 mM NaCl, 1 mM EDTA, 0.5 mM EGTA). Nuclei were disrupted using LB3 buffer (10 mM Tris-HCl pH 8.0, 200 mM NaCl, 1 mM EDTA, 0.5 mM EGTA, 0.1% NaDeoxycholate, 0.5% N-lauroylsarcosine. All lysis buffers were complemented with 1 mM EDTA, 1 mM EGTA, 1 mM DTT, 50 mM NaF, 1 mM Na_3_VO_4 _and protease inhibitors before use. The released chromatin was sonicated to obtain fragments of 200 bp using a COVARIS sonicator and immediately after sonication 0.5% Triton X-100 was added to the samples to help the solubilization of the shared DNA. Samples were spun at 10,000g for 10 minutes and half of the obtained material was incubated overnight with 5 μg HA-ChIP antibody (Abcam **Cambridge, UK**) at 4°C. The antibody molecules were pulled down using Dynal protein G magnetic beads (Invitrogen), washed and the bound material was released using Elution buffer (50 mM Tris-HCl pH 8.0, 10 mM EDTA, 1% SDS) at 65°C. The DNA-protein crosslinking was reverted by incubating the samples overnight at 65°C. The DNA was treated with RNaseA and proteinase K and extracted using a phenol-chloroform procedure. The size and the amount of the obtained DNA was confirmed prior to library preparation. Purified DNA with total amounts of 10 ng was used for sequencing library preparation using the Illumina TruSeq DNA Sample Preparation Kit v2 (Illumina, San Diego, CA, USA). The standard protocol was followed, with one modification: to accommodate for low amounts of input DNA, the adapter mix was applied in a tenfold dilution. Sequencing was performed using the Illumina HiSeq 2000 platform by the Biomedical Sequencing Facility at the CeMM Research Institute for Molecular Medicine of the Austrian Academy of Sciences. All samples were sequenced with 50 bp single-end reads and multiplexing using Illumina's third-read barcoding scheme. Initial data processing and quality control were performed using the CASAVA (Illumina) and FastQC [53] software packages. Sequencing reads were trimmed by clipping regions with low base-calling quality or adapter contamination, and the resulting quality-filtered reads were aligned to the hg19/GRCh37 assembly of the human genome using Bowtie [1]. Next, UCSC Genome Browser WIG/bigWig tracks and peak calls were established using the MACS software with default parameters - for example, minimum score 50 representing peaks at *P*-value < 1E-5. Sequencing data were submitted to the Gene Expression Omnibus database (NCBI) and assigned the identifier GSE47539.

### Statistics

In general, the statistical tests applied in the paper are indicated with the *P*-values as well as a multiple hypothesis correction according to BH [54] if necessary. The test for the binding specificities was constructed as follows: as the spectral counts do not follow a standard statistical distribution, we decided to apply nonparametric statistical methods. Furthermore, we combined the spectral counts obtained from the three different cell lines, where a given protein was not necessarily expressed at identical levels. Accordingly, we developed a permutation test based on the Wilcoxon rank sum test statistic *W *(equivalent to Mann-Whitney *U*). The three cell lines are denoted CL_x _with × = 1,2,3. Each protein *P *was tested separately. For a given nucleic acid subtype (for example, DNA) and a cell line *x*, the spectral counts of *P *in pulldowns with baits having the chosen subtype were collected in a vector *u *whereas the spectral counts for the other pulldowns were collected in *v*. A statistic $W_{CL_{x}}\left(P\right)$ (2 levels of subscripting) was computed with the R function wilcox.test comparing *u *and *v *with default parameters. We then combined the statistics of the three cell lines according to:

$$W_{tot}(P)=\frac{\sum sc_{CL_{1}}\left(P\right)W_{CL_{1}}(P)+\sum sc_{CL_{2}}(P)W_{CL_{2}}(P)+\sum sc_{CL_{3}}(P)W_{CL_{3}}(P)}{\sum sc_{CL_{1}}(P)+\sum sc_{CL_{2}}(P)+\sum sc_{CL_{3}}(P)},$$

where $\sum^{S}C_{CL_{x}}(P)$ was the sum of *P *spectral counts in CL_x_. This weighting scheme aided in eliminating the influence of cell lines with low protein abundance that could not yield significant test statistics and would otherwise mask potential significance originating from another cell line. Random permutations preserving the cell line origin of the data allowed us to estimate *P*-values for the new weighted test statistic *W_tot_*(*P*).

Binding specificity at the domain level was assessed by multiplying the *P*-values of all the identified domain-containing proteins for each subtype of nucleic acids. The *P*-value corresponding to this product was obtained by applying a theorem we published in Supplementary Information of a previous paper [55]. The determination of low complexity and disordered regions in protein sequences was realized as described in [15].

From UCSC Genome Bioinformatics [56] we downloaded reduced representation bisulfite sequencing (RRBS) data for four biological replicates of HEK293 cells that are part of the ENCODE data [32]. Genomewide YB-1 methylated cytosine affinity was tested by comparing (KS one-sided) percentages of mCG within ±150 bp windows around MACS peaks versus the percentage outside these windows in the four ENCODE HEK293 datasets. ENCODE mCG sites with coverage below 10 were discarded. The network analysis of YB-1 gene targets was realized using a human interactome composed of the data present in IntAct, BioGRID, HPRD, DIP, InnateDB, and MINT and a diffusion process named random walk with restart [57] (restart probability set at 0.3). The principle consisted of mapping YB-1 ChIP-seq peaks nearest genes (maximum 5,000 bp distance) to the interactome (206 proteins). The mapped genes were used as seeds for the random walk with identical probabilities, and after convergence to the asymptotic distribution, we added to the seed genes all the nodes that obtained an asymptotic probability at least as good as the minimum seed asymptotic probability. The largest connected component constituted the cancer-associated subnetwork. GO analysis of the full gene target lists and the subnetwork was obtained from the DAVID web site (GO FAT and clustering) [58].

## Abbreviations

AP: affinity purification; BH: Benjamini-Hochberg (multiple hypothesis correction); bp: base pair; ChIP: chromatin immunoprecipitation; ChIP-chip: chromatin immunoprecipitation on chip; ChIP-seq: chromatin immunoprecipitation sequencing; dsDNA: double-stranded DNA; FA: FERM adjacent domain; FPR: false positive rate; GO: Gene Ontology; HCDB: high confidence direct binder; KS: Kolmogorov-Smirnov; MS: mass spectrometry; NABP: nucleic acid-binding protein; NCL: nucleolin; PBS: phosphate-buffered saline; ssDNA: single-stranded DNA; ssRNA: single-stranded RNA; TPR: true positive rate.

## Competing interests

The authors declare that they have no competing interests.

## Authors' contributions

JC, GSF, and GD designed the study, analyzed the data and wrote the manuscript. TB and KLB co-designed the study. GD, TB, KH, RG, EK, IK, AM, and KLB prepared and analyzed biological samples. TD, TRB, and AS analyzed data. GFE, HL, and PB supervised part of the data analysis. All authors read and approved the final manuscript.

## Supplementary Material

Additional file 1 Supplementary information, including most of the supplementary tables (except the largest ones, which are provided in Additional files 2,3,4,5 but with descriptions in Additional file 1) and all supplementary figures. Click here for file

Additional file 2 **Supplementary Table S5**. Click here for file

Additional file 3 **Supplementary Table S8**. Click here for file

Additional file 4 **Supplementary Table S9**. Click here for file

Additional file 5 **Supplementary Table S10**. Click here for file
