# Supplementary material for: Experimental characterization of the human non-sequence-specific nucleic acid interactome
Source: Genome Biol. 2013 Jul 31;14(7):R81. doi: 10.1186/gb-2013-14-7-r81 (PMC4053969; doi:10.1186/gb-2013-14-7-r81)
Supplement: Additional file 1 — Supplementary information, including most of the supplementary tables (except the largest ones, which are provided in Additional files 2345 but with descriptions in Additional file 1) and all supplementary figures. [file gb-2013-14-7-r81-S1.PDF]

Supplementary Information

Experimental characterization of the human non sequence-specific nucleic acid interactome

Gerhard Dürnberger[1], Tilmann Bürckstümmer[1,a], Kilian Huber[1], Roberto Giambruno[1], Tobias Doerks[2], Evren Karayel[1], Thomas R Burkard[1,b], Ines Kaupe[1,c], André C. Müller[1], Andreas Schönegger[1], Gerhard F Ecker[3], Hans Lohninger[4], Peer Bork[2], Keiryn L Bennett[1], Giulio Superti-Furga[1,*], Jacques Colinge[1,*]

[1]CeMM Research Center for Molecular Medicine of the Austrian Academy of Sciences, 1090 Vienna, Austria

[2]Structural and Computational Biology Unit, EMBL - European Molecular Biology Laboratory, 69117 Heidelberg, Germany

[3]Department of Medicinal Chemistry, University of Vienna, 1090 Vienna, Austria

[4]Institute of Chemical Technologies and Analytics, Vienna University of Technology, 1060 Vienna, Austria

[*]Correspondence to: Giulio Superti-Furga or Jacques Colinge, CeMM Research Center for Molecular Medicine of the Austrian Academy of Sciences, AKH-BT 25.3, Lazarettgasse 14, 1090 Vienna, Austria. Tel.: +43 1 40160 70011; Fax: +43 1 40160 970000; Email: GSuperti-Furga@cemm.oeaw.ac.at or JColinge@cemm.oeaw.ac.at

Supplementary Table S1

	RNA	DNA	
	ss	ss	ds
T T T A A T A A T A A T T T T A A T A A T A A T A T A A T T	x	x	x
C C C A A C A A C A A C C C C A A C A A C A A C A C A A C C	x	x	x
G G G A A G A A G A A G G G G A A G A A G A A G A G A A G G	x	x	x
C C C T T C T T C T T C C C C T T C T T C T T C T C T T C C	x	x	[a]
G G G T T G T T G T T G G G G T T G T T G T T G T G T T G G	x	x	[a]
G G G C C G C C G C C G G G G C C G C C G C C G C G C C G G	x	x	x
G G G C m G C m G C m G G G G C m G C m G C m G m G C m G G		x	xx[b]
A A A A A A A A A A A A A A A A A A A A A A A A A A A A A A	x	x	
C C C C C C C C C C C C C C C C C C C C C C C C C C C C C C		x	
T T T T T T T T T T T T T T T T T T T T T T T T T T T T T T		x	
A T A T A T A T A T A T A T A T A T A T A T A T A T A T A T		x	
N N N N N N N N N N N N N N N N N N N N N N N N N N N N N N		x	

[a]equivalent to ds GA(CA) DNA

[b]hemi- and fully methylated

Supplementary Table S2. Negative control pulldowns contents.

UniProt AC	Gene name	NA/strep ratio	NA binding	UniProt AC	Gene name	NA/strep ratio	NA binding
O43707	ACTN4	0.0	FALSE	P62851	RPS25	5.4	TRUE
Q562R1	ACTBL2	0.7	FALSE	P08708	RPS17	5.5	FALSE
B0I1T2-1	MYO1G	0.8	FALSE	P62829	RPL23	5.7	FALSE
Q9ULV4	CORO1C	0.8	FALSE	P46783	RPS10	5.7	FALSE
Q96P11-2	NSUN5	0.8	FALSE	P83881	RPL36A	6.0	FALSE
P56537	EIF6	1.3	TRUE	P46776	RPL27A	6.8	TRUE
Q8IZQ5	SELH	1.5	FALSE	P62081	RPS7	6.8	TRUE
P68363	TUBA1B	1.6	FALSE	P23396	RPS3	7.5	TRUE
P26373	RPL13	2.0	TRUE	P62701	RPS4X	7.6	TRUE
Q13765	NACA	2.0	TRUE	P05164-1	MPO	7.7	FALSE
Q6P5R6	RPL22L1	2.0	FALSE	P42766	RPL35	8.0	TRUE
P62888	RPL30	2.3	TRUE	P62266	RPS23	8.0	FALSE
P61513	RPL37A	2.8	FALSE	P62269	RPS18	8.0	TRUE
P32969	RPL9	2.8	TRUE	P62273	RPS29	8.0	FALSE
O00488	ZNF593	2.8	TRUE	P63173	RPL38	8.0	TRUE
P27824	CANX	3.0	FALSE	P83731	RPL24	8.0	TRUE
P46782	RPS5	3.3	TRUE	P62277	RPS13	8.4	TRUE
P62913-1	RPL11	3.4	TRUE	P62241	RPS8	8.5	FALSE
P47914	RPL29	3.5	TRUE	P62899	RPL31	9.0	TRUE
P50914	RPL14	4.0	TRUE	P18621	RPL17	12.0	FALSE
Q8WVM0	TFB1M	4.0	TRUE	P38646	HSPA9	12.0	FALSE
Q96P11-1	NSUN5	4.0	FALSE	P61626	LYZ	12.0	FALSE
Q9NV06-1	DCAF13	4.0	FALSE	P37108	SRP14	14.5	TRUE
Q9UNQ2	DIMT1L	4.0	TRUE	P62753	RPS6	15.0	FALSE
P62861	FAU	4.1	TRUE	P16401	HIST1H1B	15.3	TRUE
P62987	UBA52	4.5	FALSE	P11142-1	HSPA8	18.0	FALSE
P39019	RPS19	4.7	TRUE	Q86V81	THOC4	18.0	TRUE
Q969Q0	RPL36AL	4.7	FALSE	Q92522	H1FX	18.0	TRUE
P60866	RPS20	4.8	TRUE	P62263	RPS14	20.7	TRUE
P62249	RPS16	4.8	TRUE	P61247	RPS3A	22.7	TRUE
P11021	HSPA5	4.8	FALSE	P49458	SRP9	23.0	TRUE
P62854	RPS26	5.0	TRUE	P35268	RPL22	25.0	TRUE
P62857	RPS28	5.0	FALSE	P62750	RPL23A	26.5	TRUE
P62847-1	RPS24	5.2	FALSE	P62917	RPL8	28.0	TRUE
P62280	RPS11	5.3	TRUE	P62244	RPS15A	36.0	TRUE
Q14512	FGFBP1	5.3	FALSE	P17844	DDX5	44.5	TRUE

Supplementary Table S3. Novel nucleic acid binding proteins identified in this study.

Uniprot AC	Gene name	Uniprot AC	Gene name	Uniprot AC	Gene name	Uniprot AC	Gene name
P61626	LYZ	Q8N442	GUF1	P60903	S100A10	Q96HY6-1	DDRGK1
O15230	LAMA5	Q8NCF5-1	NFATC2IP	Q16181-1	SEPT7	Q96MI9-1	AGBL1
Q6P1M3-1	LLGL2	Q8TAT6-1	NPLOC4	Q5T160	RARS2	Q9Y3D3	MRPS16
Q86WW8	COA5	Q96BP2	CHCHD1	Q8IY17-1	PNPLA6	A0FGR8-1	ESYT2
Q99988	GDF15	Q9BPX6-1	MICU1	Q8NBJ5	GLT25D1	O14975	SLC27A2
Q9BQP7	C20orf72	Q9HBH1	PDF	Q96CX2	KCTD12	O75165	DNAJC13
Q9H7H0-1	METTL17	Q9ULX3	NOB1	Q9BVC6	TMEM109	P51571	SSR4
Q08426	EHHADH	Q96T88	UHRF1	Q9NVI7-1	ATAD3A	Q8N3E9	PLCD3
Q52LJ0	FAM98B	Q9BXK1	KLF16	Q9UMD9-1	COL17A1	Q9BSJ8-1	ESYT1
Q8NFQ6	BPIFC	O00746	NME4	Q9Y251	HPSE	Q9BYN8	MRPS26
Q9BVC5	C2orf49	Q13268	DHRS2	Q9Y3D5	MRPS18C	Q9H0U3	MAGT1
Q9BXX2-1	ANKRD30B	Q69YN2-1	CWF19L1	Q9Y446	PKP3	Q9H0U6	MRPL18
Q9BY49-1	PECR	Q7Z4W1	DCXR	Q9Y4K1	AIM1	Q9NSI2-1	FAM207A
Q9HCM4-2	EPB41L5	Q8IWL3	HSCB	Q13555-1	CAMK2G	P05164-1	MPO
Q9P1Y5	CAMSAP3	Q92506	HSD17B8	Q9NVI7-2	ATAD3A	Q96FZ2	C3orf37
Q8N3K9	CMYA5	Q9Y3E5	PTRH2	Q2TBE0-1	CWF19L2	P27448-1	MARK3
Q9NUI1-1	DECR2	O15235	MRPS12	P30049	ATP5D	Q13303-2	KCNAB2
P12268	IMPDH2	P09543-1	CNP	Q8IYU8	EFHA1	Q14722-1	KCNAB1
Q8NFW8-1	CMAS	P23368	ME2	P24539	ATP5F1	Q3MHD2-1	LSM12
Q99856	ARID3A	P48426	PIP4K2A	P41223	BUD31	Q86YV0-1	RASAL3
Q9BW19	KIFC1	Q02809	PLOD1	Q00325-1	SLC25A3	P48741	HSPA7
Q96GY0	ZC2HC1A	Q7LGA3-1	HS2ST1	Q15019	SEPT2	O43822-1	C21orf2
O43491	EPB41L2	Q9H078-1	CLPB	Q7Z2Y8	GVINP1	Q13303-1	KCNAB2
Q6UB35-1	MTHFD1L	Q9UDY2-1	TJP2	Q9H089	LSG1	Q9H0G5	NSRP1
Q9UNX3	RPL26L1	Q9Y305-1	ACOT9	Q9P035	PTPLAD1	Q9P016-1	THYN1
O00515	LAD1	Q9BW92	TARS2	P06753-2	TPM3	P67936-1	TPM4
O43709-1	WBSCR22	Q9P2D7-1	DNAH1	O75947-1	ATP5H	Q9NQW1-1	SEC31B
O95470	SGPL1	Q17RN3-1	FAM98C	O94874	UFL1	P63010-1	AP2B1
P02751-1	FN1	Q86W50-1	METTL16	P39656	DDOST	Q96CW1-1	AP2M1
P08708	RPS17	P36957	DLST	P42695	NCAPD3	O94916-1	NFAT5
P10646-1	TFPI	Q02218	OGDH	Q00325-2	SLC25A3	P00367	GLUD1
P49006	MARCKSL1	Q14512	FGFBP1	Q53H96	PYCRL	P48436	SOX9
Q2TAY7	SMU1	P43003	SLC1A3	Q8WVX9	FAR1	P20160	AZU1
Q2TB90-1	HKDC1	P50416-1	CPT1A	Q969G5	PRKCDBP	Q4LEZ3	AARD
Q53SF7-1	COBLL1	P51659	HSD17B4	Q96DP5	MTFMT		

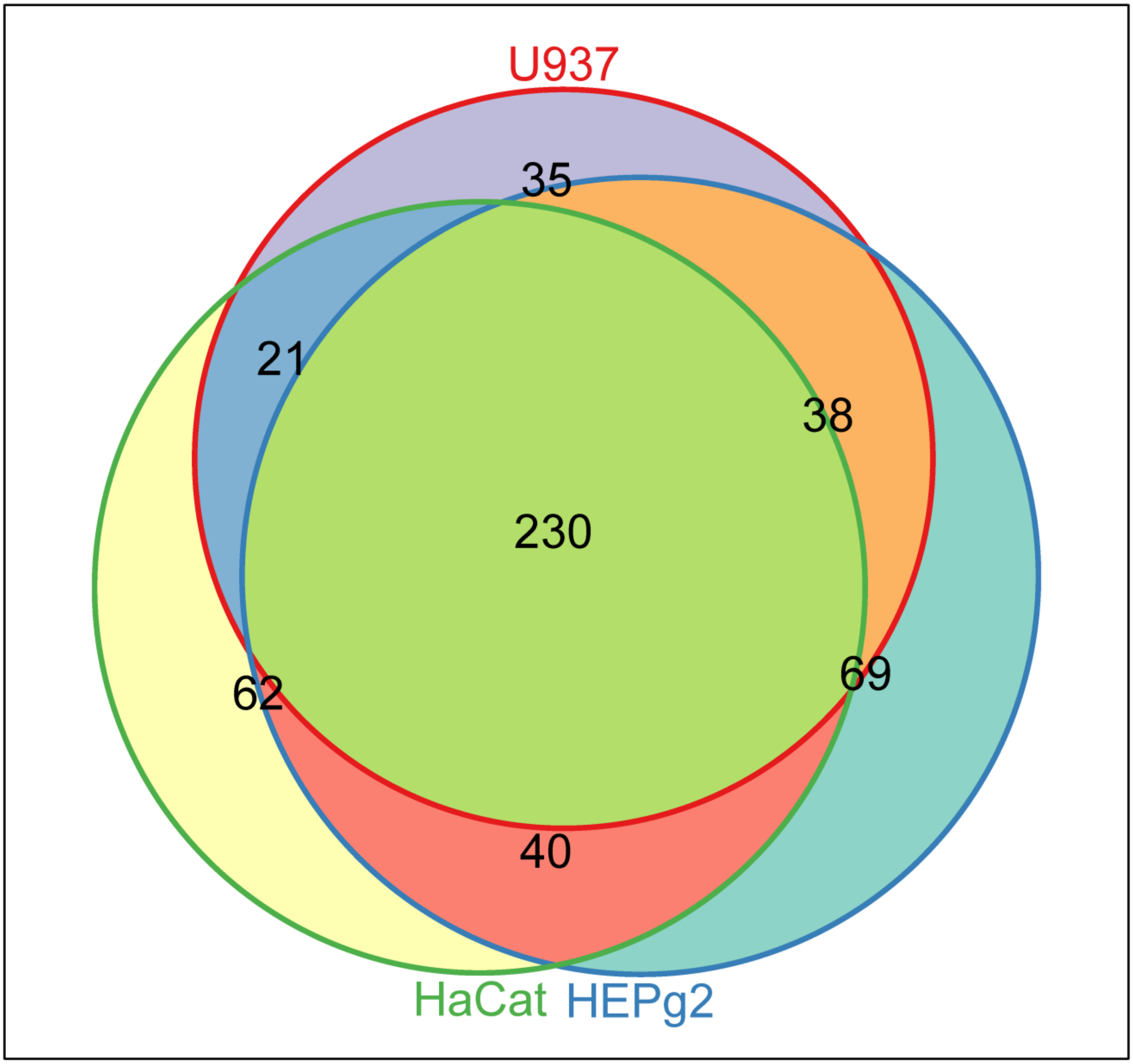

Supplementary Figure S1. The distribution of the 495 known NABPs identified across the three cell lines is almost uniform, there is no bias for a given cell line.

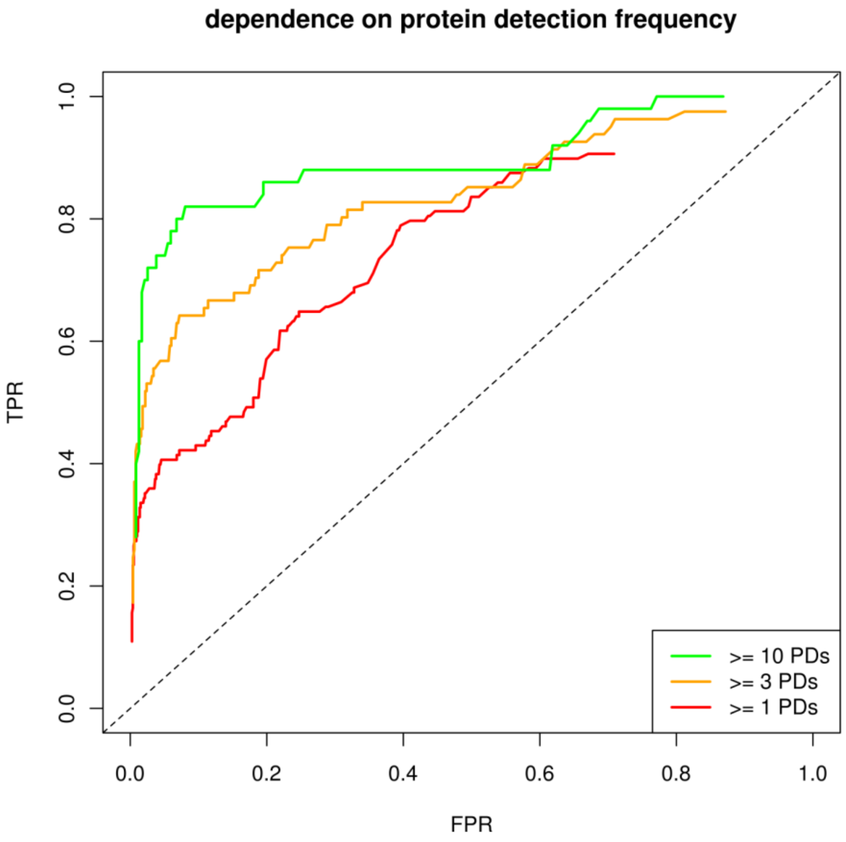

Supplementary Figure S2. Nucleic acid binding inference true positive rate (TPR) in dependence of the amount of MS data available (statistical test presented in the manuscript). The amount of data is represented here by the number of pulldowns (PDs) were a given protein was detected, i.e. the green curve represents TPRs versus false positive rates (FPRs) of proteins identified in at least 10 PDs out of the 75 pulldowns realized (not counting the 3 negative controls). We clearly see that the more data available the better the test sensitivity at any imposed FPR.

Supplementary Table S4. GO MF analysis of the 746 likely direct NA binders identified in the study (DAVID web tool, GO MF level 3, Huang et al., Nature Protoc., 2009).

Term	Count	% in data	PValue	Benjamini-Hochberg corrected
GO:0003723 RNA binding	293	40.0	1.24E-216	1.62E-214
GO:0003677 DNA binding	192	26.2	1.01E-15	6.49E-14
GO:0003743 translation initiation factor activity	24	3.3	1.84E-15	8.18E-14
GO:0008135 translation factor activity, nucleic acid binding	29	4.0	6.15E-15	1.98E-13
GO:0016875 ligase activity, forming carbon-oxygen bonds	14	1.9	1.85E-07	4.81E-06
GO:0016817 hydrolase activity, acting on acid anhydrides	64	8.7	1.34E-05	2.90E-04

Supplementary Table S5. Nucleic acid sub-class specificities with P-value < 0.05. RNA interactome indicates presence in the Castello et al. (Cell, 2012) protein list. At the 0.05 significance level, on the basis of selected specificities for which a GO annotation of an entry in Castello's list was available, we found that the inferred DNA specificities had a true positive rate (TPR) of 23.0% and a false positive rate (FPR) of 2.8%, whereas inferred RNA specificities had a TPR of 18.7% and a FPR of 1.6%. Performing the selection at the 0.01 significance level, the TPR and FPR scaled nicely: DNA TPR 17.8%, FPR 1.2%; RNA TPR 10.1%, FPR 0.4%.

The table is provided as an independent spreadsheet file.

Supplementary Figure S3. GO BP term significance in the various sets of proteins inferred to bind specifically one or several sub-classes of nucleic acids. We observe the clear separation between biological processes enriched in inferred DNA and RNA binding proteins. Color log scale: red=P<1E-15, light yellow=P<0.01, gray=P≥0.01.

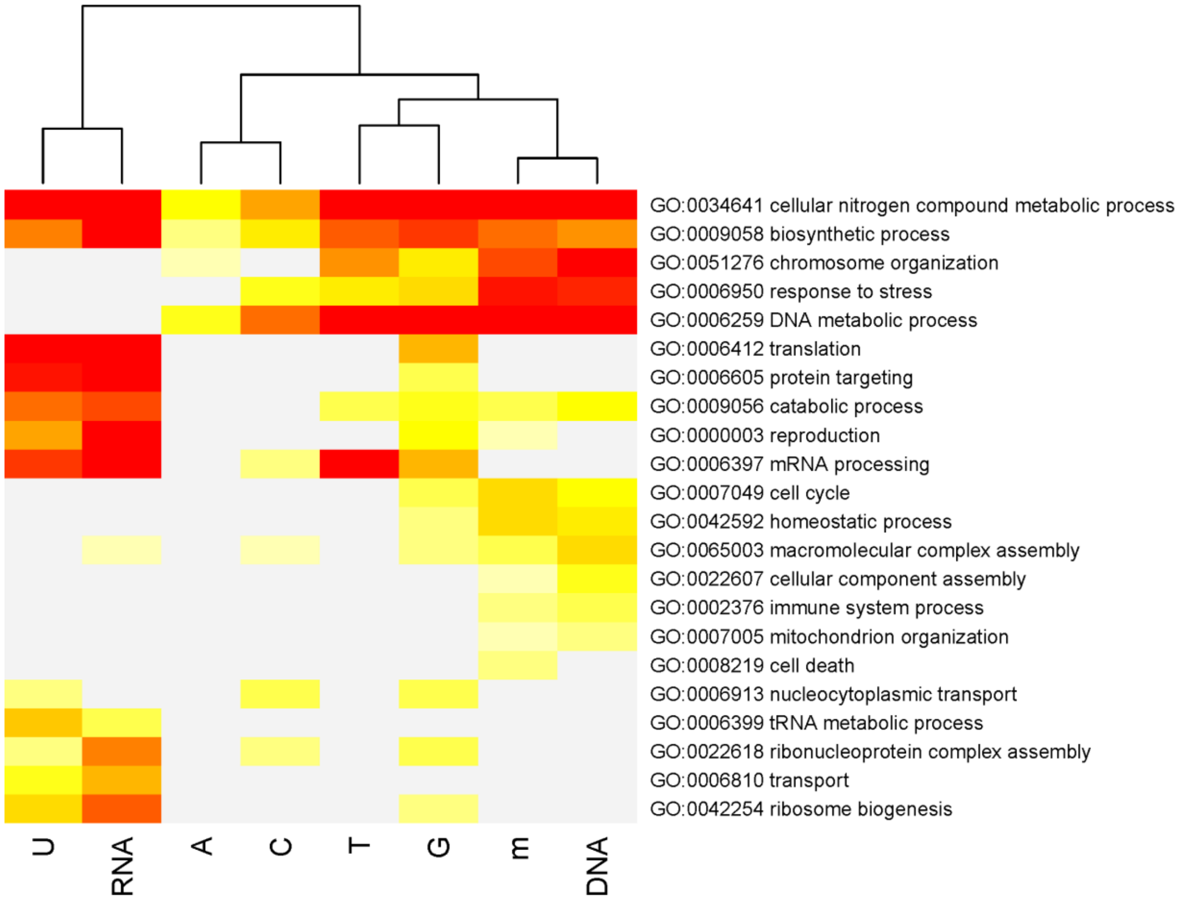

Supplementary Figure S4. GO CC term significance in the various sets of proteins inferred to bind specifically one or several sub-classes of nucleic acids. As it was the case in Figure S2, we observe the clear separation between molecular functions enriched in inferred DNA and RNA binding proteins. Color log scale: red=P<1E-15, light yellow=P<0.01, gray=P≥0.01.

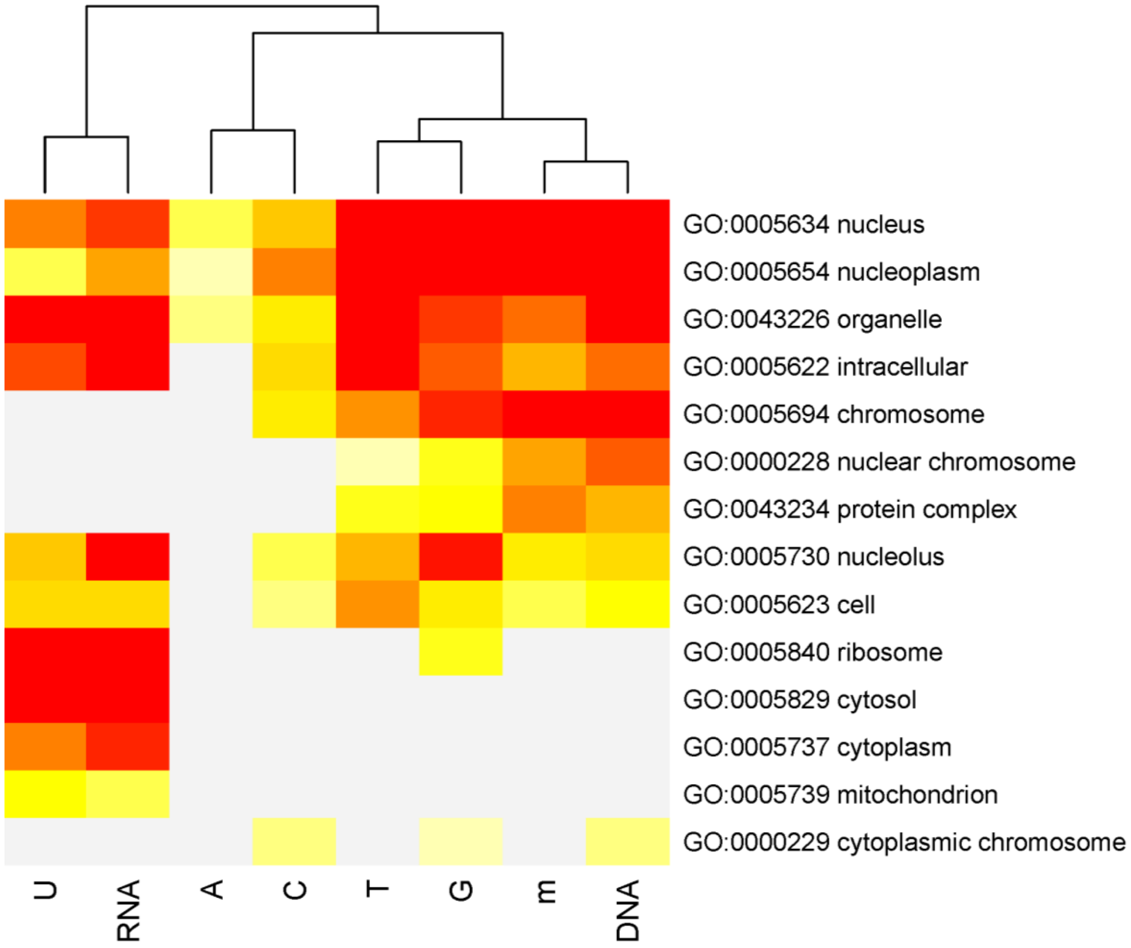

Supplementary Figure S5. Statistical test for methylated cytosine percentage at YB-1 positions of binding versus the rest of the genome. YB-1 binding was determined in HEK293 cells by ChIP-seq by us. Significant P-values (p) are underlined in red ($p<0.05$, Kolmogorov-Smirnov one-sided). The quantity n refers to the number of sites that passed our criteria (on line Materials and Methods). The four samples Stanford 1&2 and UW 1&2 (all HEK293 cells) were independently analyzed as part of the ENCODE projects (bisulfate sequencing).

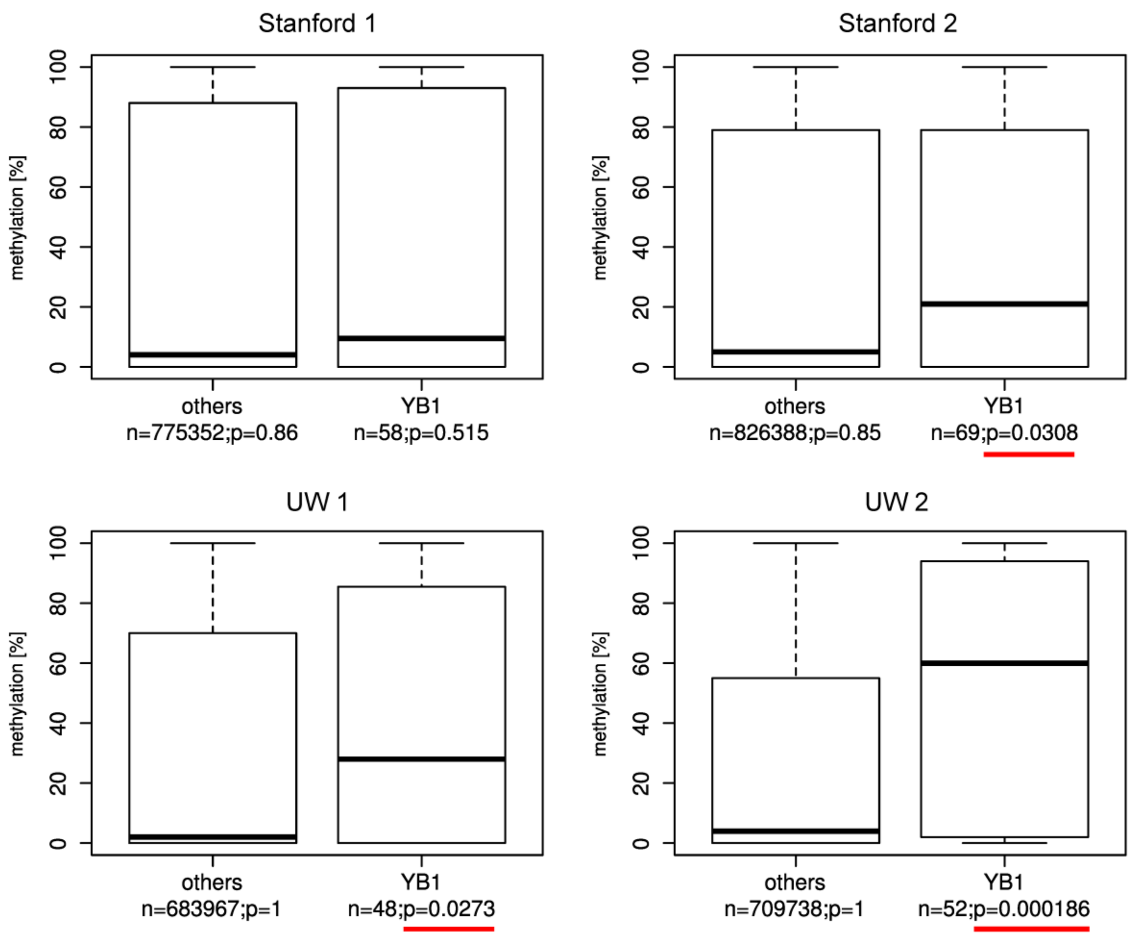

Supplementary Figure S6. UCSC Genome browser local view. YB-1 ChIP-seq data are labeled "Layered H3K27Ac" and we can observe the correspondence with the 4 ENCODE bisulfite-seq datasets (tracks HEK293 St 1&2 and UW 1&2). Also note the match with methylated CpGs in the additional ENCODE cell lines GM12878, H1-hESC, K562, HeLa-S3, HepG2, and HUVEC at the bottom of the figure.

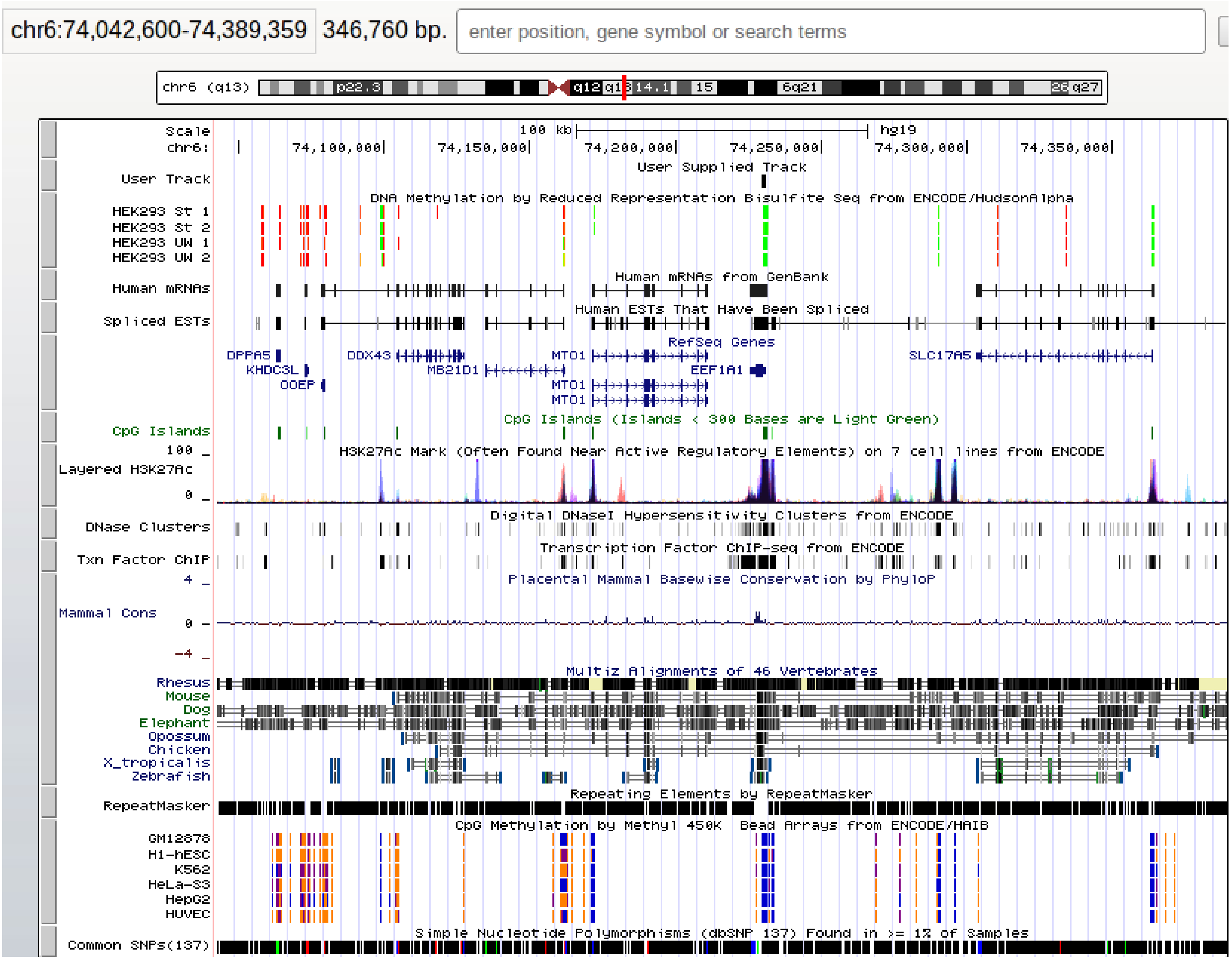

Supplementary Table S6. Domains that were enriched in the NABP devoid of Pfam domain associated with nucleic acid binding affinity.

Pfam ID	Domain name	P-value	P-value (BH corr.)	Identified proteins UniProt AC / Gene name
PF08736	FA	5.9E-6	2.0E-3	Q9HCM4-2 / EPB41L5<br>Q43491 / EPB41L2<br>Q9Y4F1-1 / FARP1<br>Q9H4G0-2 / EPB41L1
PF10239	DUF2465	2.6E-5	8.6E-3	Q52LJ0 FAM98B<br>Q8NCA5 / FAM98A<br>Q17RN3-1 / FAM98C
PF00106	adh_short	7.2E-5	2.4E-2	Q9BY49-1 / PECR<br>Q9NUI1-1 / DECR2<br>Q16698 / DECR1<br>Q13268 / DHRS2<br>Q6YN16-1 / HSDL2<br>Q7Z4W1 / DCXR<br>Q92506 / HSD17B8<br>P51659 / HSD17B4
PF03914	CBF	8.6E-5	2.8E-2	Q9BVI4 / NOC4L<br>Q8WTT2 / NOC3L
PF04676<br>PF04677	CwfJ_C_2<br>CwfJ_C_1	8.6E-5	2.8E-2	Q69YN2-1 / CWF19L1<br>Q2TBE0-1 / CWF19L2
PF04900	Fcf1	8.6E-5	2.8E-2	Q9Y324 / FCF1<br>Q9BRU9-1 / UTP23
PF09532<br>PF09542<br>PF12701	DFDF<br>FFD_TFG<br>LSM14	8.6E-5	2.8E-2	Q8ND56-1 / LSM14A<br>Q9BX40-1 / LSM14B

Supplementary Table S7. Genetic associations of the NABPs found in this study that were not annotated in GO previously, Associations were obtained from the Genetic Association Database (Zhang et al., BMC Med Genomics, 2010).

ID	Gene Name	Disease Class	GENETIC_ASSOCIATION_DB_DISEASE
O00139	kinesin heavy chain member 2A	PSYCH,	schizophrenia,
O95470	sphingosine-1-phosphate lyase 1	NEUROLOGICAL,	Alzheimer's Disease,
P02751	fibronectin 1	CANCER,CARDIOVASCULAR,OTHER,PSYCH,	cryoglobulinemic fibronectin lymphoma,heart disease, ischemic,lung cancer,schizophrenia,systemic sclerosis,
P05164	myeloperoxidase	AGING,CANCER,NEUROLOGICAL,etc.	agranulocytosis,Alzheimer's Disease,anti-neutrophil cytoplasmic antibodies kidney failure, cancer, etc.
P06753	tropomyosin 3	OTHER,	nemaline myopathy,
P07195	lactate dehydrogenase B	PSYCH,	panic disorder,
P07355	annexin A2, pseudogenes 1&3	METABOLIC,	osteonecrosis,
P08195	solute carrier family 3, member 2	PHARMACOGENOMIC,	melphalan pharmacokinetics melphalan side effects,
P09543	2',3'-cyclic nucleotide 3' phosphodiesterase	CARDIOVASCULAR,PSYCH,	hypertension,schizophrenia,
P09622	dihydrolipoamide dehydrogenase	NEUROLOGICAL,	Alzheimer's Disease,
P10646	tissue factor pathway inhibitor	CARDIOVASCULAR,HEMATOLOGICAL,METABOLIC ,	bleeding complications,stroke,thromboembolism, venous,thromboembolism
P23368	malic enzyme 2, NAD(+)-dependent	NORMALVARIATION,	epilepsy, idiopathic generalized,
P34897	serine hydroxymethyltransferase 2	NEUROLOGICAL,	neural tube defects,
P36957	dihydrolipoamide S-succinyltransferase	DEVELOPMENTAL,	Alzheimer's Disease,Alzheimer's disease; Wernicke-Korsakoff syndrome,
P43003	solute carrier family 1, member 3	NEUROLOGICAL,	schizophrenia,
P48426	phosphatidylinositol-5-phosphate 4-kinase, type II, alpha	PSYCH,	bipolar disorder schizophrenia,schizophrenia,schizophrenia; bipolar disorder,
P48436	SRY (sex determining region Y)-box 9	PSYCH,	hypospadias,
P50416	carnitine palmitoyltransferase 1A (liver)	DEVELOPMENTAL,	diabetes, type 2 hepatic lipid content insulin,hepatitis C, chronic,left ventricular hypertrophy,obesity,
P51398	death associated protein 3	CARDIOVASCULAR,INFECTION,METABOLIC,	asthma,
P60903	S100 calcium binding protein A10	IMMUNE,	depressive disorder, major,
P62937	similar to TRIMCyp; peptidylprolyl isomerase A	PSYCH,	HIV,
Q00059	transcription factor A, mitochondrial	INFECTION,	Alzheimer's Disease,Alzheimer's disease Parkinson's disease,endurance performance,
Q02809	procollagen-lysine 1, 2-oxoglutarate 5-dioxygenase 1	NEUROLOGICAL	bone density,longer forms of a repetitive sequence,
Q05682	caldesmon 1	METABOLIC,OTHER,	diabetes, type 1,nephropathy in other diseases,
Q13555	calcium/calmodulin-dependent protein kinase II gamma	IMMUNE,	Alzheimer's Disease,
Q99988	growth differentiation factor 15	NEUROLOGICAL,	colorectal cancer,
Q9BXK1	Kruppel-like factor 16	CANCER,	diabetes, type 2,
Q9UEY8	adducin 3 (gamma)	METABOLIC,	arterial stiffness,hypertension,
Q9UMD 9	collagen, type XVII, alpha 1	CARDIOVASCULAR,	bullous pemphigoid,periodontitis,
Q9Y251	heparanase	IMMUNE,CANCER,CARDIOVASCULAR	Hodgkin's disease leukemia, myeloid multiple myeloma myelodysplastic syndrome, stroke

Supplementary Table S8. The 206 nearest genes at maximum 5000 bp distance to YB-1 ChIP-seq peaks. The table is provided as an independent spreadsheet file.

Supplementary Figure S7. The 206 genes found to be targeted by YB-1 in a ChIP-seq experiment contained a subset of 73 genes that form a protein interaction network strongly associated with KEGG cancer pathways ($P<3x10^{-4}$). BY-1 targeted genes are in green and strongly interconnected additional genes from the human protein interactome identified by means of a random walk analysis (Köhler et al., 2008).

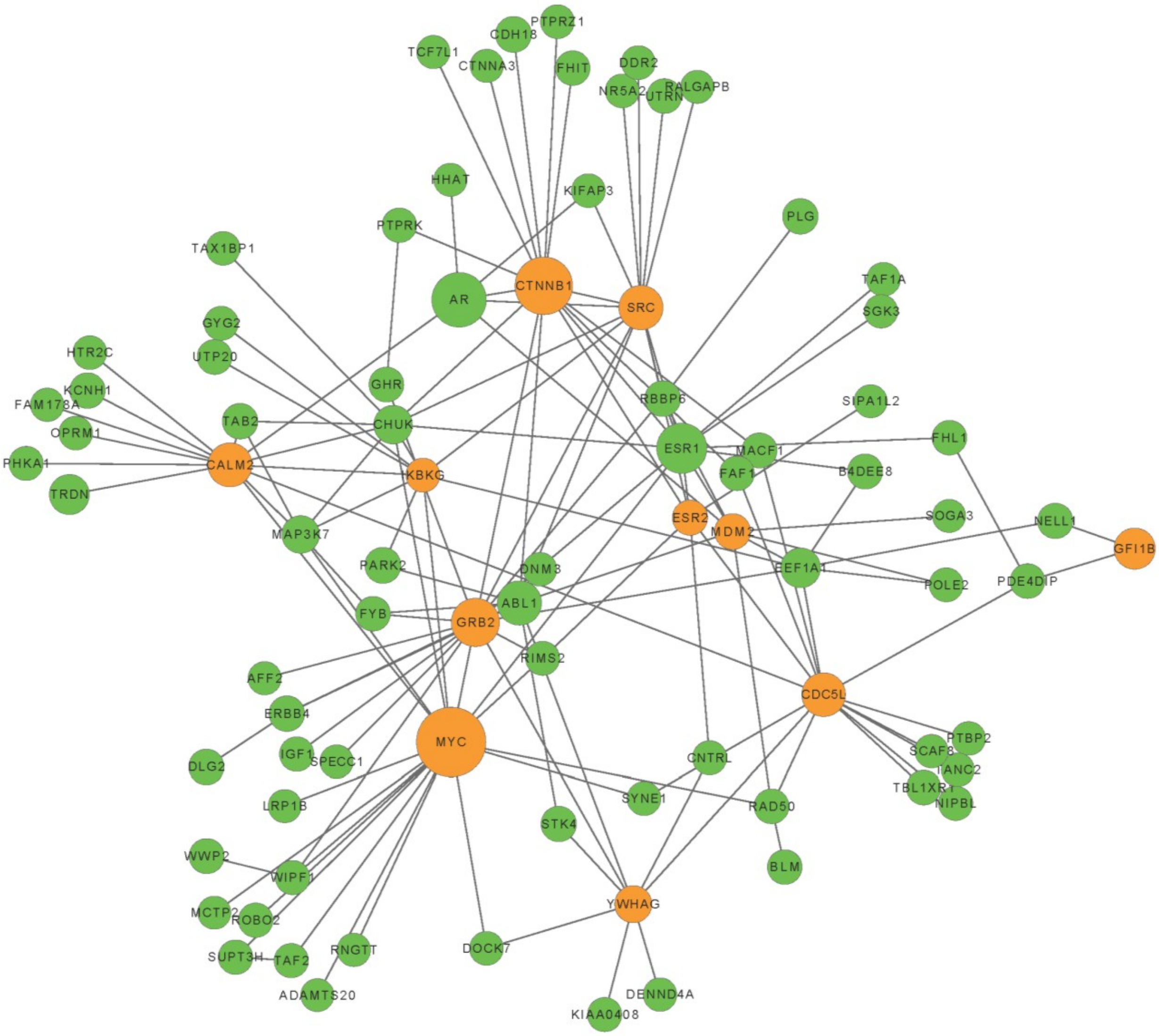

Supplementary Table S9. Lists of all the identified proteins with spectral counts reported for each synthetic bait. The table is provided as independent spreadsheet files.
